# Emotional expressions of old faces are perceived as more positive and less negative than young faces in young adults

**DOI:** 10.3389/fpsyg.2015.01276

**Published:** 2015-08-26

**Authors:** Norah C. Hass, Erik J. S. Schneider, Seung-Lark Lim

**Affiliations:** Department of Psychology, University of Missouri-Kansas City, Kansas City, MO, USA

**Keywords:** emotions, age, facial perception, facial expressions, perceptual decision

## Abstract

Interpreting the emotions of others through their facial expressions can provide important social information, yet the way in which we judge an emotion is subject to psychosocial factors. We hypothesized that the age of a face would bias how the emotional expressions are judged, with older faces generally more likely to be viewed as having more positive and less negative expressions than younger faces. Using two-alternative forced-choice perceptual decision tasks, participants sorted young and old faces of which emotional expressions were gradually morphed into one of two categories—“neutral vs. happy” and “neutral vs. angry.” The results indicated that old faces were more frequently perceived as having a happy expression at the lower emotional intensity levels, and less frequently perceived as having an angry expression at the higher emotional intensity levels than younger faces in young adults. Critically, the perceptual decision threshold at which old faces were judged as happy was lower than for young faces, and higher for angry old faces compared to young faces. These findings suggest that the age of the face influences how its emotional expression is interpreted in social interactions.

## Introduction

Interpreting the emotional state of others is an important part of our daily life. Our judgments of others’ emotions (e.g., happy or angry) may even determine whether we choose to interact with them or not. Thus, assessing the emotions of others can alert us to valuable social information, such as whether that person is approachable, friendly, trustworthy, or threatening (McArthur and Baron, 1983; Oosterhof and Todorov, 2009; Willis et al., 2011). In assessing emotions, we naturally look to facial expressions and can make rapid social judgments even from only brief exposures (Willis and Todorov, 2006). However, the perceptual judgment of emotions is a subjective psychological process and can be easily influenced by various psychosocial variables, often resulting in misleading information (e.g., judging someone as angry when he or she is not). Not surprisingly, the perceiver’s emotional state is one of these critical psychosocial factors. For example, depressive symptoms have been implicated in a greater bias toward perceiving negative emotions as well as an enhanced sensitivity toward perceiving angry emotions (Liu et al., 2012). Studies also indicate that implicit attitude or prejudice for a certain group can bias emotional perception in faces, causing someone to more easily label a face as angry when it belongs to the stereotyped group than when it belongs to their own group (Hugenberg and Bodenhausen, 2003). Interestingly, the perceiver’s age also impacts emotional judgment of others (Mill et al., 2009). In general, older adults tend to label angry, anxious, sad, or disgust emotions less frequently or perceived them less negatively than younger adults (Keightley et al., 2007; Czerwon et al., 2011).

In addition to the variables highlighted above, the age of faces can be another psychosocial factor that can influence judgment of emotions. Compared to young neutral faces, old neutral faces generally tend to be judged as less positive in several domains such as attractiveness, likability, and distinctiveness (Wernick and Manaster, 1984; Ebner, 2008), which may reflect an aging-related stereotype. Given that the interpretation of another’s emotional state can impact subsequent interactions, it is critical to better understand how the age of a face can influence the perceived emotion. If the age of a face systematically changes the emotion it is thought to be expressing, then significant implications for older adults could likely exist. Since research suggests an implicit negative aging stereotype to be pervasive in age-related attitudes (Kite and Johnson, 1988; Hummert et al., 2002), it is even more crucial to examine the influence facial age may have on the subjective perceptual decision of varying facial expressions.

Previous research that has examined the role of age in the perception of emotions has heavily focused on behavioral differences between young and old age groups. A meta-analytic study showed that older adults tend to have more difficulties and be less accurate in recognizing basic emotions in facial expressions as compared to younger adults (Ruffman et al., 2008). While young adults show generally very high levels of accuracy across different types of facial expressions (often with a ceiling effect), older adults tend to demonstrate a difficulty in identifying emotions, in particular negative emotions (Keightley et al., 2007; Sullivan et al., 2007; Ebner and Johnson, 2009). Research from this perspective provides useful information in understanding aging-related changes in the ability to accurately recognize and attend to emotional expressions. However, it does not directly address the question of whether the age of the facial image being judged plays a role in how it is perceived. Shifting the focus from what past research has emphasized, we are interested in whether manipulating the age of the stimuli impacts the emotion it is judged to possess.

The age of a face can play a critical role in decoding emotional expressions (Folster et al., 2014). In general, previous studies reported that emotional facial expressions of old faces are less accurately perceived or less confidently categorized compared to young faces, irrespective of the underlying emotional expressions (Borod et al., 2004; Ebner et al., 2011, 2012, 2013; Riediger et al., 2011; Hess et al., 2012). Both face-related factors (e.g., lower expressivity, age-related changes in faces) and observer-related factors (e.g., negative attitude toward old people, different visual scan path) have been suggested as potential underlying mechanisms responsible for the decreased accuracy of emotional judgment for old faces (see Folster et al., 2014, for a review). However, the majority of studies have employed only prototype faces for each emotional expression category (e.g., 100% angry face, 100% happy face, 100% neutral face). Thus, except accuracy information for the prototype faces categorization, they could not answer how the perceptual decision threshold (i.e., the emotional intensity level at which perceptual decision shifts from neutral to emotion that expresses) would be systematically modulated by the age of a face. Given that emotional expression in social interaction usually occurs along a continuum rather than at the extreme levels, it is important to understand the systematic effect of the age of a face on the decision threshold of affective facial perception, which has not been investigated yet.

The present study aims to examine whether the age of a face has a systematic impact on the *subjective perceptual decision threshold* of emotional judgment on a continuum of emotional expression (0 ∼ 100% relative to each identity’s maximum emotional expression). Since real-life interactions generally include a range of emotions and emotional intensities, it is important to systematically explore the decision thresholds at which individuals’ shift their categorization of a face from saying it is not emotional to saying it expresses an emotion. Also, because young adults are well known to be accurate at identifying emotions in faces compared to older adults (Keightley et al., 2007; Sullivan et al., 2007; Ruffman et al., 2008; Ebner and Johnson, 2009), our experiment was designed to focus on a young adult sample to explore the perceptual decision threshold changes by the age of faces while minimizing a potential effect of accuracy differences.

Based on previous literature (Ruffman et al., 2008; Ebner et al., 2011; Folster et al., 2014), we hypothesize that participants will identify positive (happy) or negative (angry) emotional expressions of older faces differently compared to the same level of emotional expressions in young faces across systematically varied intensities of emotional expression. Specifically, we expect that older faces will be more likely to be perceived as happy compared to young faces (i.e., lower perceptual decision threshold for positive emotion), but less likely to be perceived as angry compared to young faces (i.e., higher perceptual decision threshold for negative emotion). In social interactions, emotional judgment provides important information for approach vs. avoidance decision-making (e.g., decision to interact or not in an initial encounter). Because older adults are often perceived as less threatening or viewed as high on warmth and thus are judged as more approachable in social interactions (Cuddy and Fiske, 2002; Ebner et al., 2011; Willis et al., 2015), we speculated that ambiguous emotional expressions of older faces are more likely to be judged as expressing positive emotion and less likely to be judged as expressing negative emotion by young adults.

In order to investigate our research hypothesis, we designed two similar two-alternative forced-choice (2AFC) tasks which asked individuals to categorize young and old facial images on a binary basis as either “Happy” or “Neutral” for the first task, or “Angry” or “Neutral” for the second task. This paradigm was chosen because it allows for perceptual decision threshold parameters to be estimated through non-linear psychometric curve fits and has been successfully implemented in previous emotion research (Lim and Pessoa, 2008; Lee et al., 2012; Lim et al., 2014; Weston et al., 2015). Further, varying the emotions on a continuum is expected to increase the external validity of our study, since the facial expression of emotions naturally varies in strength.

## Materials and Methods

### Participants

Participants consisted of 33 healthy college students with a mean age of 26.1 years (SD = 8.5 years; 23 females; 23 Caucasian, 3 Hispanic, 4 African American, 2 Asian, and 1 other) that were recruited though the Psych Pool online research participant recruitment system at the University of Missouri-Kansas City (UMKC). Two additional participants were recruited but excluded from analyses due to unreliable behavioral data (i.e., continuously giving one response throughout all trials). Participants received course credits in compensation for individually completing the in-laboratory experiment. The study protocol was reviewed and approved by UMKC’s Institutional Review Board. Participants provided their written informed consent before participation.

### Experimental Task

**FIGURE 1:**
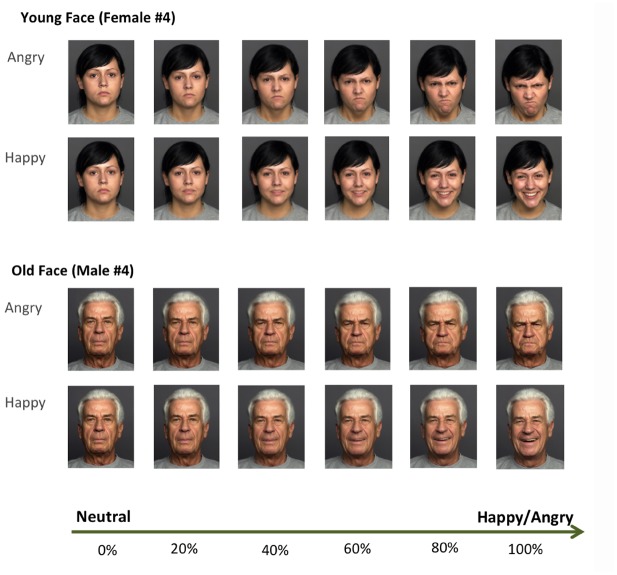
**Exemplar facial stimuli used for the age judgment task.** Total eight identities (two young and two old male identities, and two young and two old female identities; only two identities are shown here for examples) were used in the main experiment. Emotional expression and age of facial stimuli were manipulated by using morphing software. Faces have emotion gradients ranging from 0% (neutral emotion) to 100% (full emotion; either happy or angry) by increments of 20%.

Participants performed a novel, computerized affective perception task developed to test our research hypotheses about the effect of facial age on the perceptual judgment of emotional expressions. The neutral (0%), happy (100%), and angry (100%) face stimuli used in this task were taken from the FACES database (Ebner et al., 2010), and emotional expression gradients for each facial identity were then parametrically manipulated in increments of 20% (i.e., across six levels) using FantaMorph software (Abrosoft, Lincoln, NE, USA). Experimental stimuli included eight different, Caucasian identities (two old males, two old females, two young males, and two young females), ranging across six emotional intensity levels from “neutral” to “happy” (in 20% intervals of increasingly happy expressions) in one condition, and “neutral” to “angry” (in 20% intervals of increasingly angry expressions) in the other condition (see Figure 1, for examples).

**FIGURE 2:**
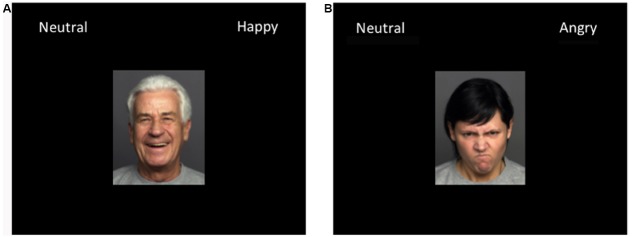
**(A)** Sample screen of the neutral-happy judgment task. (B) Sample screen of the neutral-angry judgment task. Participants were asked to make perceptual judgments about the emotion of faces (neutral vs. happy; neutral vs. angry) in a two-alternative forced-choice procedure.

The affective perception task was divided into two different types of blocks. The first type of block tested how participants perceived neutral or happy facial expressions of young and older adults (positive affect block), while the second block measured how participants perceived neutral or angry facial expressions of young and older adults (negative affect block). The order of blocks was counterbalanced across participants to control for potential order effects. In both blocks, participants made a series of two-alternative forced-choice emotion judgments for young and old faces at each level of emotional expressions, randomized in their presentation. For the positive affect block trials, participants were asked to state whether they thought the emotion of the facial image shown on the computer screen was “Neutral” or “Happy” by pressing the keyboard key that corresponded to the respective category (Figure 2A). For negative affect block trials, participants were asked to judge whether they thought a facial image was “Neutral” or “Angry” (Figure 2B). Each face stimulus (300 by 375 pixels) was presented in the center of the screen for a period of 100 ms followed by a blank screen presented for 2 s during which the participant was instructed to either press the “i” or “e” keyboard button to record the response. The brief stimulus presentation was employed in our experimental paradigm to eliminate or minimize the occurrence of deliberate eye saccades (Pessoa et al., 2005), as similarly done in previous perceptual decision making studies (Lim and Pessoa, 2008; Dagovitch and Ganel, 2010; Hole and George, 2011; Lee et al., 2012; Lynn et al., 2012; Marneweck et al., 2013; Seirafi et al., 2013; Lim et al., 2014; Nikitin and Freund, 2015; Weston et al., 2015). Also, it has been shown that people can make rapid social judgments even from only brief (e.g., 100 ms) exposures of faces (Willis and Todorov, 2006) without significantly increasing reliability when given more time. For half of the participants, the “i” key indicated the “Neutral” category with the word “Neutral” displayed in the upper right corner of the screen, and the “e” key indicated the “Happy” (positive affect block) or “Angry” (negative affect block) category with the appropriate word displayed in the upper left corner of the screen. For the other half of the participants, the key button mapping was flipped. Participants were told to categorize the facial stimuli as quickly and as accurately as possible. Two seconds were allotted for a response before the word “MISS” was presented in red to indicate that the participant took over 2000 ms to respond. After each response was given, a yellow crosshair was shown for 500 ms in the center indicating that the response was recorded. A white crosshair was then presented for a variable amount of time (between 1 and 2 s, with 50 ms variations) before the next face was shown to direct focus to the middle of the screen. Within each type of block, stimulus presentation order was fully randomized, such that participants saw the emotion expression levels and the faces of both ages in random order. After each block was completed, the participant saw text on the screen informing him/her that the section was completed before moving on to the next block. SuperLab software (Cedrus Corporation, San Pedro, CA, USA) was used to present and collect data for each participant. This software assessed the decision choice (whether neutral or angry/happy) and reaction times for each decision. The affective perception task contained 480 trials for each type of block (2 age groups × 6 emotion intensity levels × 40 repetitions) and took approximately 1 h to complete.

### Statistical Analysis and Psychometric Curve Fitting

Relating the proportion of “Happy” or “Angry” decisions to different levels of emotional intensities of the younger and older faces, we applied a non-linear psychometric curve-fitting approach to our data that has been successfully employed in previous emotion research (Lim and Pessoa, 2008; Lee et al., 2012; Lim et al., 2014). Similar to these studies, psychometric curves were fitted for each experimental condition by using the Naka-Rushton contrast response model (Albrecht and Hamilton, 1982; Sclar et al., 1990) with an *OLS* (ordinary least square) criterion.

(1) $$response=\frac{R_{\max}*C^{n}}{C^{n}+C_{50}^{n}}+M$$

Here, *response* represents the proportion of “Happy” (positive affect block) or “Angry” (negative affect block) decisions, *C* is the emotional intensity levels of the young and older faces (contrast: 0 ∼ 100% happy or 0 ∼ 100% angry in 20% increments), *C*_50_ is the stimulus intensity at which the response is half-maximal (also called the “threshold” or “point of subjective equality: PSE”), *n* is the exponent parameter that represents the slope of the function, *R_max_* is the asymptote of the response function, and *M* is the response at the lowest stimulus intensity. Given that the proportion of “Happy” or “Angry” decisions to “Neutral” decisions (minimum 0; maximum 1) was used, the *R_max_* parameter was constrained to be equal to or less than 1 and the *M* parameter was constrained to be equal to or larger than 0. For the pooled data, we fitted psychometric curves separately for each type of experimental condition (young happy faces, old happy faces, young angry faces, and old angry faces). Curve fitting was done with GraphPad Prism software (GraphPad Software, La Jolla, CA, USA).

**FIGURE 3:**
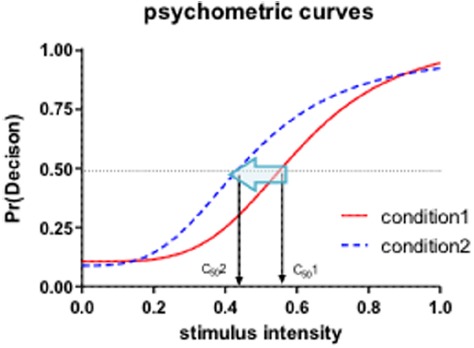
**Psychometric curves (Naka-Rushton contrast response model).** X-axis represents stimulus intensity and Y-axis represents response probability. In our experiments, the stimulus intensity represents the incremental increase of emotional expressions (neutral to happy; neutral to angry) of morphed faces and the response represents the proportion of happy or angry decisions. The C_50_ or PSE (point of subjective equality) parameter indicates the perceptual decision threshold. A leftward shift of the psychometric curve would constitute evidence for decreased perceptual threshold.

We hypothesized that the age of facial stimuli would influence affective perception on emotional expressions of both happiness and anger by systematically changing the decision threshold (*PSE*) that is represented by *C*_50_ parameter. As illustrated in Figure 3, the changes of threshold are often described by a leftward shift (decrease of decision threshold) or a rightward shift (increase of decision threshold) of psychometric curves by the contrast gain model in visual perception research (Reynolds and Chelazzi, 2004; Huang and Dobkins, 2005; Carrasco, 2006) and has been reported in previous studies of affective perception of facial stimuli (Lim and Pessoa, 2008; Lee et al., 2012; Lim et al., 2014).

#### Repeated-Measures Analysis of Variance (ANOVA)

To test our research hypotheses, the task-irrelevant effect of the age of faces on the affective judgment (happy or angry expressions) of facial stimuli was systematically examined by employing repeated-measures ANOVAs and non-linear psychometric curve fitting approaches. We applied Greenhouse–Geisser corrections for repeated-measures statistics and Bonferroni corrections for *post-hoc* tests.

## Results

### Affective Perception Task

**FIGURE 4:**
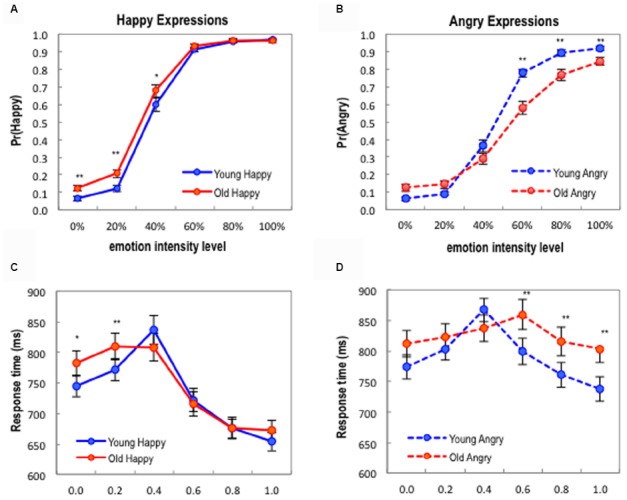
**(A)** Probabilities of happy decisions as a function of emotion intensity and facial age. **(B)** Probabilities of angry decisions as a function of emotion intensity and facial age. **(C)** Response times of happy decisions. (D) Response times of angry decisions. Error bars denote the standard error of the mean. *p < 0.05; **p < 0.01 (Bonferroni corrections applied).

First, for happy and angry expression judgment tasks, we performed separate 2 (AGE: Young faces, Old faces) by 6 (EMOTION INTENSITY: 0 ∼ 100% in 20% increments) repeated-measures ANOVAs on the behavioral data of proportions of happy or angry decisions to neutral decisions. Means and standard deviations are shown in Table 1. The ANOVA result on happy decisions revealed a significant two-way interaction effect of AGE × EMOTION INTENSITY, *F*(5,160) = 6.67, *p* < 0.001, ${\text{η}}_{\text{p}}^{\text{2}}$ = 0.17. We also observed main effects of AGE, *F*(1,32) = 22.71, *p* < 0.001, ${\text{η}}_{\text{p}}^{\text{2}}$ = 0.42, and EMOTION INTENSITY, *F*(5,160) = 672.58, *p* < 0.001, ${\text{η}}_{\text{p}}^{\text{2}}$ = 0.96. To clarify this interaction effect through simple effect analyses, we performed a series of paired *t*-tests for each level of EMOTION INTENSITY. As shown in Figure 4A, at the low levels of happy expressions (0, 20, and 40%), old faces were more frequently perceived as having a happy expression than young faces, *t*(32) = 4.63, *p* < 0.01, *d* = 0.78; *t*(32) = 4.19, *p* < 0.01, *d* = 0.71; *t*(32) = 2.92, *p* < 0.05, *d* = 0.37, whereas there was no significant difference between ages at the high levels of happy expressions (60, 80, and 100%), all *p* > 0.05. The ANOVA result on angry decisions also revealed a significant two-way interaction effect of AGE × EMOTION INTENSITY, *F*(5,160) = 25.13, *p* < 0.001, ${\text{η}}_{\text{p}}^{\text{2}}$ = 0.44, as well as main effects of AGE, *F*(1,32) = 13.62, *p* < 0.001, ${\text{η}}_{\text{p}}^{\text{2}}$ = 0.30, and EMOTION INTENSITY, *F*(5,160) = 544.64, *p* < 0.001, ${\text{η}}_{\text{p}}^{\text{2}}$ = 0.95. Interestingly, subsequent simple effect analyses on angry expressions revealed findings that were somewhat opposite to those of the happy expressions. As shown in Figure 4B, old faces were less frequently perceived as angry than young faces were at the high levels of angry expressions (60, 80, and 100%), *t*(32) = –6.96, *p* < 0.01, *d* = 1.10; *t*(32) = –5.38, *p* < 0.01, *d* = 0.82; *t*(32) = –3.81, *p* < 0.05, *d* = 0.70, whereas there was no significant difference at 20 and 40% levels, all *p* > 0.05. At 0% level (neutral faces), old faces were more frequently perceived as with an angry expression, *t*(32) = 4.16, *p* < 0.05, *d* = 0.68. Because the proportion data (between 0 and 1) did not show normal distributions across all levels, we further checked the robustness of our findings with additional non-parametric statistics. The signed ranks Wilcoxon tests with Bonferroni corrections showed similar results as before (0, 20, and 40% happy expressions, *Z* = –3.72, *p* < 0.01, *Z* = –3.69, *p* < 0.01, *Z* = –2.69, *p* < 0.05; 0, 60, 80, and 100% angry expressions, *Z* = –4.19, *p* < 0.001, *Z* = –4.70, *p* < 0.001, *Z* = –4.25, *p* < 0.001, *Z* = –3.63, *p* < 0.01).

**TABLE 1:** **Means and standard deviations of the proportion of happy and angry decisions**.

**Face type**	**Emotion intensity level of morphed faces**					
	0%	20%	40%	60%	80%	100%
Young happy	0.063 (0.064)	0.122 (0.101)	0.599 (0.226)	0.913 (0.081)	0.958 (0.049)	0.969 (0.044)
Old happy	0.125 (0.090)	0.207 (0.136)	0.678 (0.209)	0.931 (0.065)	0.962 (0.060)	0.960 (0.051)
Young angry	0.061 (0.055)	0.090 (0.060)	0.364 (0.193)	0.778 (0.139)	0.895 (0.103)	0.920 (0.078)
Old angry	0.126 (0.125)	0.143 (0.138)	0.288 (0.183)	0.581 (0.211)	0.770 (0.190)	0.846 (0.127)

Second, we performed similar 2 (AGE) by 6 (EMOTION INTENSITY) repeated-measures ANOVAs on the response time data of happy or angry decisions. Means and standard deviations of the response times are shown in Table 2. All response time data revealed normal distributions in the Kolmogorov–Smirnov test (all *p*-values > 0.05). For the happy expression judgment task, the ANOVA result showed a significant interaction effect of AGE × EMOTION INTENSITY, *F*(5,160) = 5.86, *p* < 0.001, ${\text{η}}_{\text{p}}^{\text{2}}$ = 0.167. Main effects of AGE, *F*(1,32) = 4.40, *p* < 0.05, ${\text{η}}_{\text{p}}^{\text{2}}$ = 0.12, and EMOTION INTENSITY, *F*(5,160) = 59.97, *p* < 0.001, ${\text{η}}_{\text{p}}^{\text{2}}$ = 0.65, were also significant. Again, we performed a series of paired *t*-tests for each level of EMOTION INTENSITY to clarify the interaction effect. As shown in Figure 4C, at the low levels of happy expressions (0 and 20%), perceptual decision time for old faces was slower than young faces, *t*(32) = 3.05, *p* < 0.05, *d* = 0.34; *t*(32) = 3.68, *p* < 0.01, *d* = 0.34, whereas there was no significant difference at the other levels of happy expressions (40, 60, 80, and 100%), all *p* > 0.05. Also, the ANOVA result on the response time of the angry judgment task revealed a significant interaction effect, *F*(5,160) = 7.44, *p* < 0.001, ${\text{η}}_{\text{p}}^{\text{2}}$ = 0.19, a main effect of AGE, *F*(1,32) = 22.24, *p* < 0.001, ${\text{η}}_{\text{p}}^{\text{2}}$ = 0.41, and a main effect of EMOTION INTENSITY, *F*(5,160) = 11.72, *p* < 0.001, ${\text{η}}_{\text{p}}^{\text{2}}$ = 0.27. Similar subsequent simple effect analyses showed that perceptual decision time for old faces were slower than young faces at the higher levels of angry expressions (60, 80, and 100%), *t*(32) = 4.21, *p* < 0.01, *d* = 0.46; *t*(32) = 3.60, *p* < 0.01, *d* = 0.44; *t*(32) = 4.68, *p* < 0.01, *d* = 0.53, whereas there was no significant difference at the lower levels of angry expressions (0, 20, and 40%), all *p* > 0.05 (see Figure 4D). Combined with the decision data, the response time results suggest that the increase of happy decisions on old faces at the low level of emotional intensity and the decrease of angry decisions on old faces at the high level of emotional intensity were associated with additional cognitive processes (and thus, longer response times) in the affective perception of old faces.

**TABLE 2:** **Means and standard deviations of the response time in milliseconds**.

**Face type**	**Emotion intensity level of morphed faces**					
	0%	20%	40%	60%	80%	100%
Young happy	775 (100)	771 (102)	837 (135)	722 (110)	676 (90)	655 (88)
Old happy	782 (119)	809 (123)	808 (131)	716 (116)	677 (104)	672 (96)
Young angry	775 (155)	804 (105)	867 (109)	799 (124)	761 (115)	738 (114)
Old angry	812 (127)	824 (122)	837 (127)	860 (141)	816 (137)	846 (134)

### Perceptual Decision Threshold

**FIGURE 5:**
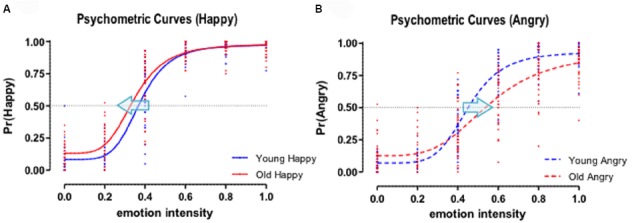
**(A)** Psychometric curves for happy decisions. **(B)** Psychometric curves for angry decisions. Psychometric curves were fitted by using the Naka-Rushton response function. A leftward shift (blue arrow) represents the decrease of perceptual decision threshold (C_50_ parameter) for old facial expressions compared to young facial expressions, whereas a rightward shift represents the increase of perceptual decision threshold for old facial expressions compared to young facial expressions for each respective emotion.

As stated earlier we hypothesized that the age of target face stimuli would systematically influence the decision threshold of affective perception of facial expressions. More specifically, we postulated that young adult participants would show a decreased perceptual threshold for positive emotion (i.e., happy faces), but they would show an increased perceptual threshold for negative emotion (i.e., angry faces) for older faces. In our 2AFC tasks, the perceptual decision threshold or *PSE* that determines binary responses (i.e., neutral vs. happy; neutral vs. angry), was indexed by estimating *C*_50_ parameters (i.e., the emotional intensity values in the x-axis that produce 50% happy or angry decisions) from choice data. The estimated best-fit values and standard errors of the Naka-Rushton contrast response model are shown in Table 3. For happy expressions, the means of the *C*_50_ parameter for young faces and old faces were 0.376 (SE = 0.009) and 0.347 (SE = 0.011), respectively. On the other hand, the means of the *C*_50_ parameter for young angry faces and old angry faces were 0.452 (SE = 0.010) and 0.556 (SE = 0.038). All *C*_50_ parameters showed normal distributions in the Kolmogorov–Smirnov test (all *p*-values > 0.05). A model comparison nested *F*-test that assumes equal *C*_50_ parameters for old and young happy faces showed a significant result, *F*(1,388) = 3.98, *p* < 0.05, indicating that the perceptual threshold for happy expressions was decreased for old faces compared to young faces (see a leftward shift of Figure 5A). A model comparison nested *F*-test of *C*_50_ parameters for angry faces also showed a significant result, *F*(1,388) = 13.39, *p* < 0.01, indicating the perceptual threshold for angry expressions was increased for old faces compared to young faces, *t*(32) = 4.82, *p* < 0.01, *d* = 0.93 (see a rightward shift of Figure 5B). In other words, as the *C*_50_ parameters indicate, participants required only 34.7% emotion intensity level to make a happy decision for old faces, while they required 37.6% emotion intensity level for young faces. The average decrease for old happy faces across participants was –3.2% (95% *CI*: –0.9 ∼ –5.4%). For angry decisions, participants required 55.6% emotion intensity level to make an angry decision for old faces, while they required 45.2% emotion intensity level for young faces. The average increase of old angry faces across participants was +12.0% (95% *CI*: +6.9 ∼ +17.1%). These results imply that young participants more sensitively perceive positive emotional expressions of old faces compared to young faces (i.e., a positive decision bias for old happy faces), while they less sensitively perceive negative emotional expressions of old faces compared to young faces (i.e., a negative decision bias for old angry faces).

**TABLE 3:** **Means and standard errors of best-fit values of psychometric curve fit parameters**.

**Face type**	***C*_50_**	***R*_max_**	***n***	***M***
Young happy	0.376 (0.009)	0.894 (0.029)	5.027 (0.876)	0.084 (0.018)
Old happy	0.347 (0.011)	0.850 (0.029)	4.462 (0.574)	0.131 (0.020)
Young angry	0.452 (0.010)	0.864 (0.028)	5.337 (0.598)	0.069 (0.015)
Old angry	0.556 (0.038)	0.802 (0.084)	3.744 (0.780)	0.126 (0.023)

## Discussion

American society is becoming older, with an estimated 19.7% of the population over the age of 60 and the average lifespan of men and women in America calculated to be 76.4 and 81.2 years, respectively (The Economist, 2014). Because of this, it is becoming increasingly important to understand the psychological mechanisms behind the social perceptions for older adults, particularly the perceptions younger adults hold, in consideration of older adults’ overall well-being. In this study, we looked specifically at how emotions may be perceived differently in younger compared to older faces.

The primary purpose of this study was to explore how age, as a task-irrelevant variable, influences the perceptual judgment of positive and negative emotional expressions in young adults. We specifically examined the impact of facial age on the 2AFC categorization of emotions that varied along a continuum. This allowed us to determine whether the age (young vs. old) of facial stimuli systematically impacted the subjective perceptual decision threshold in affective perception of faces. Our hypothesis was that older faces would be judged more likely as showing a positive emotion, specifically, happiness, than for younger faces, but that older faces would be judged less likely for negative, angry facial judgments. This was supported through the interaction found between facial age and the emotional decision. Specifically, the subjective perceptual threshold for categorizing a face as having a happy compared to a neutral expression was significantly lower for older faces than for younger faces, but this threshold for judging a face as neutral or angry was significantly higher for older faces. In other words, old faces were more frequently labeled “happy” than young faces at the 0, 20, and 40% emotion levels. In a similar way, old faces were less frequently labeled “angry” compared to young faces at the 60, 80, and 100% emotion levels. These results would appear to complement each other since in the low levels of positive emotionality, the old faces were more often judged as having a happy emotional expression (i.e., a leftward horizontal shift of the psychometric curve; Figure 5A), whereas at the high levels of negative emotionality the old faces were less likely to be judged as angry compared to young faces with the same levels of emotional intensity (i.e., a rightward horizontal shift of the psychometric curve; Figure 5B). Overall, these results demonstrate that the task-irrelevant stimuli age is capable of influencing subjective, emotional judgments.

The finding that old faces are more frequently judged as happy and as angry at the low-middle and high-middle emotional gradients, respectively, would appear to be contrary to previous literature at first glance, which suggests an overarching negative evaluation or low preference of old “neutral” faces compared to young “neutral” faces (Wernick and Manaster, 1984; Ebner, 2008). However, judging old emotional faces as happy, sooner, but as angry, later, on a continuum of emotional expression, would imply a positive perceptual decision bias (i.e., shifts of decision threshold) for older “emotional” faces in young adult participants, particularly in *intermediate* levels of emotional intensities. Also, an asymmetric perceptual decision bias for positive and negative emotional expressions cannot be fully explained by own age group bias (Voelkle et al., 2012), which states that individuals tend to be more accurate in judging qualities (e.g., age, prototype emotional expressions) of others who are *within* their own age group. Furthermore, the own age group bias for accuracy does not provide any specific predications about the perceptual decision threshold shifts in intermediate levels of emotional expressions. Thus, the own age group bias does not suffice as an explanation for why older faces were judged as happy sooner and angry later in an asymmetric way. Similarly, if age-related changes in physical features (e.g., wrinkles or skin color) or familiarity (i.e., more frequent encounter for own age groups; Bartlett and Fulton, 1991) existed, they would predict the shifts toward the same direction (i.e., decrease or increase of decision threshold for happy and angry expressions), not asymmetrical differences between positive and negative emotional expressions as we found in our data.

Our perceptual decision results were readily echoed with the reaction time data. The shifts of perceptual decision biases for older faces were accompanied with increased reaction times in both emotional conditions. At the low levels of happy expressions (0 and 20%), perceptual decision time for old faces was longer than for young faces, and at the higher levels of angry expressions (60, 80, and 100%) perceptual decision time for old faces was longer than for young faces. This almost directly mirrors the decision biases, which found old faces more frequently labeled as happy at the 0, 20, and 40% emotional levels but less frequently as angry at the 60, 80, and 100% levels. Given the relationship between longer reaction times and the perceptual decision biases mentioned above, it appears as though more cognitive processing resources may have been involved in these threshold cases, thus resulting in a slower decision. This highlights the differential influence that the age of faces had on the processing of emotions. Intuitively, one would expect the extreme cases—the very low and very high emotions of one’s own age group—to be overall judged more easily and quickly than facial expressions of the other age group. However, our reaction time results revealed asymmetric interactions modulated by the age of a face. Thus, it appears that age was indeed attended to and differentially modulated the processing and judging of emotional expressions. Furthermore, the increased response time for the asymmetric shifts of the decision threshold for positive and negative emotions expressions could not be simply explained by the gaze pattern differences for old and young faces (i.e., longer length of gaze time in facial images of one’s own age) found in previous research (Ebner et al., 2011).

We recognize several limitations to our study. First, we chose to use real, photographed faces manipulated across emotion levels, which have been widely used in aging research (Ebner et al., 2010). This choice was made with the intent of increasing external validity, but unintentional and uncontrolled differences of facial stimulus identities might exist. For example, there might be uncontrolled differences in the strengths of expressed emotional intensities across the identities used (both young and old) in our study. However, it should be noted that our emotional intensity levels were manipulated equally relative to maximum expressed emotional intensity for each identity (i.e., 100% happy and 100% angry), which allows us to interpret the decision threshold parameters (C_50_ relative to 100% emotion) meaningfully regardless of face identities. Using purely computer-generated faces may have better controlled for variability in face identities and the expressivity thereof, but it also would likely have impacted the realism of the faces. The lack of ethnic variability in faces beyond Caucasian suggests that further validation with more ethnically representative images would strengthen the results. However, when we compared the decision threshold changes between Caucasian subjects (*n* = 23) and non-Caucasian subjects (*n* = 10), no significant difference of observed effects was found, *t*(31) = –0.81, *p* = 0.42 for happy expression; *t*(31) = 0.72, *p* = 0.46 for angry expression.

Our experiment employed a 100 ms visual stimulus presentation for each facial image. This brief visual stimulus duration for the perceptual decision-making task was chosen to control for deliberate eye saccades or scan pattern differences between old and young faces as described in the Methods. However, real-life social interactions typically occur on a much longer time scale, which may potentially limit the ecological validity of our study. Thus, in the future it would be important to replicate or extend our study with longer stimulus duration or video presentation in order to strengthen the ecological validity of our experimental findings.

In addition, our study consisted of an arguably restricted sample (33 college students). Given the impact of participant age on emotional expression perception in other research (Keightley et al., 2007; Ebner, 2008; Ebner and Johnson, 2009; Voelkle et al., 2012), it would be informative to further validate these findings across a wider age (particularly with older adults) and demographic range of participants in future studies. However, our study, as the first of its kind, did not include an older adult sample because we were concerned about a potential confounding issue regarding emotion recognition accuracy differences between young and old age groups. When further considering the possibility of an “own-age” (Voelkle et al., 2012) or “own-group” (Thibault et al., 2006; Young and Hugenberg, 2010) bias, that is, an advantage of young participants being more accurate judging young faces and less accurate labeling old faces in our experimental context, it seems unlikely—although cannot be fully ruled out—that the significant asymmetrical shifts of decision threshold were due to lower accuracy in perceiving the expression. The findings of this study, while only intended to be generalized to young adults as we investigated, provide new information on the ways in which the facial age of a stimulus can bias the decision threshold for the emotional judgment thereof.

## Conclusion

The findings of our research add new information to existing research on age and emotional perception. They illuminate the moderating role that age plays with regards to the judgment of happy and angry facial expressions. The impact that age has on how one is perceived emotionally possesses social implications for how others may interaction with an individual. Older adults who are more quickly perceived to be happy may then be seen as more socially welcoming and approachable for interaction. In the same light, if older adults are less quickly viewed as being angry as compared to young adults, they may also be judged socially to be less threatening or dangerous. Overall, the findings of this study shed new light on how older adults are perceived and suggest future research in order to further understand the relation between emotional perception and social judgments when age is a moderating variable.

### Conflict of Interest Statement

The authors declare that the research was conducted in the absence of any commercial or financial relationships that could be construed as a potential conflict of interest.

## References

[B1] Albrecht D. G. Hamilton D. B. (1982). Striate cortex of monkey and cat: contrast response function. J. Neurophysiol. 48, 217–237. 7119846 10.1152/jn.1982.48.1.217

[B2] Bartlett J. C. Fulton A. (1991). Familiarity and recognition of faces in old age. Mem. Cogn. 19, 229–238. 10.3758/BF03211147 1861609

[B3] Borod J. C. Yecker S. A. Brickman A. M. Moreno C. R. Sliwinski M. Foldi N. S. (2004). Changes in posed facial expression of emotion across the adult life span. Exp. Aging Res. 30, 305–331. 10.1080/03610730490484399 15371098

[B4] Carrasco M. (2006). “Covert attention increases contrast sensitivity: psychophysical, neurophysiological and neuroimaging studies,” in Visual Perception. Part I. Fundamentals of Vision: Low and Mid-Level Processes in Perception—Progress in Brain Research, eds Martinez-Conde S. Macknik S. L. Martinez L. M. Alonso J. M. Tse P. U. (Amsterdam: Elsevier), 33–70. 10.1016/S0079-6123(06)54003-8 17010702

[B5] Cuddy A. J. C. Fiske S. T. (2002). “Doddering but dear: process, content, and function in stereotyping of older persons,” in Ageism: Stereotyping and Prejudice Against Older Persons, ed. Nelson T. D. (Cambridge, MA: The MIT Press), 3–36.

[B6] Czerwon B. Luttke S. Werheid K. (2011). Age differences in valence judgments of emotional faces: the influence of personality traits and current mood. Exp. Aging Res. 37, 503–515. 10.1080/0361073X.2011.619468 22091579

[B7] Dagovitch Y. Ganel T. (2010). Effects of facial identity on age judgments: evidence from repetition priming. Exp. Psychol. 57, 390–397. 10.1027/1618-3169/a000047 20178927

[B8] Ebner N. C. (2008). Age of face matters: age-group differences in ratings of young and old faces. Behav. Res. Methods 40, 130–136. 10.3758/BRM.40.1.130 18411535

[B9] Ebner N. C. He Y. Johnson M. K. (2011). Age and emotion affect how we look at a face: visual scan patterns differ for own-age versus other-age emotional faces. Cogn. Emot. 25, 983–997. 10.1080/02699931.2010.540817 21614704 PMC3339265

[B10] Ebner N. C. Johnson M. K. (2009). Young and older emotional faces: are there age group differences in expression identification and memory? Emotion 9, 329–339. 10.1037/a0015179 19485610 PMC2859895

[B11] Ebner N. C. Johnson M. K. Fischer H. (2012). Neural mechanisms of reading facial emotions in young and older adults. Front. Psychol. 3:223. 10.3389/fpsyg.2012.00223 22798953 PMC3394436

[B12] Ebner N. C. Johnson M. R. Rieckmann A. Durbin K. A. Johnson M. K. Fischer H. (2013). Processing own-age vs. other-age faces: neuro-behavioral correlates and effects of emotion. Neuroimage 78, 363–371. 10.1016/j.neuroimage.2013.04.029 23602923 PMC3684564

[B13] Ebner N. C. Riediger M. Lindenberger U. (2010). FACES—a database of facial expressions in young, middle-aged, and older women and men: development and validation. Behav. Res. Methods 42, 351–362. 10.3758/BRM.42.1.351 20160315

[B14] Folster M. Hess U. Werheid K. (2014). Facial age affects emotional expression decoding. Front. Psychol. 5:30. 10.3389/fpsyg.2014.00030 24550859 PMC3912746

[B15] Hess U. Adams R. B. Jr. Simard A. Stevenson M. T. Kleck R. E. (2012). Smiling and sad wrinkles: age-related changes in the face and the perception of emotions and intentions. J. Exp. Soc. Psychol. 48, 1377–1380. 10.1016/j.jesp.2012.05.018 23144501 PMC3491992

[B16] Hole G. George P. (2011). Evidence for holistic processing of facial age. Vis. Cogn. 19, 585–615. 10.1080/13506285.2011.562076

[B17] Huang L. Dobkins K. R. (2005). Attentional effects on contrast discrimination in humans: evidence for both contrast gain and response gain. Vision Res. 45, 1201–1212. 10.1016/j.visres.2004.10.024 15707928

[B18] Hugenberg K. Bodenhausen G. V. (2003). Facing prejudice: implicit prejudice and the perception of facial threat. Psychol. Sci. 14, 640–643. 10.1046/j.0956-7976.2003.psci_1478.x 14629699

[B19] Hummert M. L. Garstka T. A. O’Brien L. T. Greenwald A. G. Mellott D. S. (2002). Using the implicit association test to measure age differences in implicit social cognitions. Psychol. Aging 17, 482–495. 10.1037/0882-7974.17.3.482 12243389

[B20] Keightley M. L. Chiew K. S. Winocur G. Grady C. L. (2007). Age-related differences in brain activity underlying identification of emotional expressions in faces. Soc. Cogn. Affect. Neurosci. 2, 292–302. 10.1093/scan/nsm024 18985135 PMC2566756

[B21] Kite M. E. Johnson B. T. (1988). Attitudes toward older and younger adults: a meta-analysis. Psychol. Aging 3, 233–244. 10.1037/0882-7974.3.3.233 3268264

[B22] Lee T. H. Choi J. S. Cho Y. S. (2012). Context modulation of facial emotion perception differed by individual difference. PLoS ONE 7:e32987. 10.1371/journal.pone.0032987 22431992 PMC3303876

[B23] Lim S. L. Bruce A. S. Aupperle R. L. (2014). The influence of a working memory task on affective perception of facial expressions. PLoS ONE 9:e111074. 10.1371/journal.pone.0111074 25347772 PMC4210225

[B24] Lim S. L. Pessoa L. (2008). Affective learning increases sensitivity to graded emotional faces. Emotion 8, 96–103. 10.1037/1528-3542.8.1.96 18266519

[B25] Liu W. H. Huang J. Wang L. Z. Gong Q. Y. Chan R. C. (2012). Facial perception bias in patients with major depression. Psychiatry Res. 197, 217–220. 10.1016/j.psychres.2011.09.021 22357354

[B26] Lynn S. K. Zhang X. Barrett L. F. (2012). Affective state influences perception by affecting decision parameters underlying bias and sensitivity. Emotion 12, 726–736. 10.1037/a0026765 22251054 PMC3489023

[B27] Marneweck M. Loftus A. Hammond G. (2013). Psychophysical measures of sensitivity to facial expression of emotion. Front. Psychol. 4:63. 10.3389/fpsyg.2013.00063 23431121 PMC3576623

[B28] McArthur L. Z. Baron R. M. (1983). Toward an ecological theory of social perception. Psychol. Rev. 90, 215–238. 10.1037/0033-295X.90.3.215

[B29] Mill A. Allik J. Realo A. Valk R. (2009). Age-related differences in emotion recognition ability: a cross-sectional study. Emotion 9, 619–630. 10.1037/a0016562 19803584

[B30] Nikitin J. Freund A. M. (2015). Adult age differences in frequency estimations of happy and angry faces. Int. J. Behav. Dev. 39, 266–274. 10.1177/0165025414542838

[B31] Oosterhof N. N. Todorov A. (2009). Shared perceptual basis of emotional expressions and trustworthiness impressions from faces. Emotion 9, 128–133. 10.1037/a0014520 19186926

[B32] Pessoa L. Padmala S. Morland T. (2005). Fate of unattended fearful faces in the amygdala is determined by both attentional resources and cognitive modulation. Neuroimage 28, 249–255. 10.1016/j.neuroimage.2005.05.048 15993624 PMC2427145

[B33] Reynolds J. H. Chelazzi L. (2004). Attentional modulation of visual processing. Annu. Rev. Neurosci. 27, 611–647. 10.1146/annurev.neuro.26.041002.131039 15217345

[B34] Riediger M. Voelkle M. C. Ebner N. C. Lindenberger U. (2011). Beyond “happy, angry, or sad?”: age-of-poser and age-of-rater effects on multi-dimensional emotion perception. Cogn. Emot. 25, 968–982. 10.1080/02699931.2010.540812 21432636

[B35] Ruffman T. Henry J. D. Livingstone V. Phillips L. H. (2008). A meta-analytic review of emotion recognition and aging: implications for neuropsychological models of aging. Neurosci. Biobehav. Rev. 32, 863–881. 10.1016/j.neubiorev.2008.01.001 18276008

[B36] Sclar G. Maunsell J. H. Lennie P. (1990). Coding of image contrast in central visual pathways of the macaque monkey. Vision Res. 30, 1–10. 10.1016/0042-6989(90)90123-3 2321355

[B37] Seirafi M. De Weerd P. De Gelder B. L. (2013). Emotion categorization does not depend on explicit face categorization. J. Vis. 13, 12. 10.1167/13.2.12 23397037

[B38] Sullivan S. Ruffman T. Hutton S. B. (2007). Age differences in emotion recognition skills and the visual scanning of emotion faces. J. Gerontol. B Psychol. Sci. Soc. Sci. 62, P53–P60. 10.1093/geronb/62.1.p53 17284558

[B39] Thibault P. Bourgeois P. Hess U. (2006). The effect of group-identification on emotion recognition: the case of cats and basketball players. J. Exp. Soc. Psychol. 42, 676–683. 10.1016/j.jesp.2005.10.006

[B40] The Economist (2014). The Economist Pocket World in Figures: 2015 Edition. London: Profile Books Ltd.

[B41] Voelkle M. C. Ebner N. C. Lindenberger U. Riediger M. (2012). Let me guess how old you are: effects of age, gender, and facial expression on perceptions of age. Psychol. Aging 27, 265–277. 10.1037/a0025065 21895379

[B42] Wernick M. Manaster G. J. (1984). Age and the perception of age and attractiveness. Gerontologist 24, 408–414. 10.1093/geront/24.4.408 6479655

[B43] Weston T. D. Hass N. C. Lim S.-L. (2015). The effect of sad facial expressions on weight judgment. Front. Psychol. 6:417. 10.3389/fpsyg.2015.00417 25914669 PMC4392295

[B44] Willis J. Todorov A. (2006). First impressions: making up your mind after a 100-ms exposure to a face. Psychol. Sci. 17, 592–598. 10.1111/j.1467-9280.2006.01750.x 16866745

[B45] Willis M. L. Palermo R. Burke D. (2011). Judging approachability on the face of it: the influence of face and body expressions on the perception of approachability. Emotion 11, 514–523. 10.1037/a0022571 21668104

[B46] Willis M. L. Windsor N. A. Lawson D. L. Ridley N. J. (2015). Situational context and perceived threat modulate approachability judgements to emotional faces. PLoS ONE 10:e0131472. 10.1371/journal.pone.0131472 26121528 PMC4488138

[B47] Young S. G. Hugenberg K. (2010). Mere social categorization modulates identification of facial expressions of emotion. J. Pers. Soc. Psychol. 99, 964–977. 10.1037/a0020400 20919774

